# Bariatric endoscopic-surgical therapies for NAFLD. Should they be considered viable options among current treatments?

**DOI:** 10.3389/fendo.2022.1026444

**Published:** 2022-11-29

**Authors:** Eva Juárez-Hernández, Alain P. Velázquez-Alemán, Graciela Castro-Narro, Misael Uribe, Iván López-Méndez

**Affiliations:** ^1^ Translational Research Unit, Medica Sur Clinic & Foundation, Mexico City, Mexico; ^2^ Hepatology and Transplants Unit, Medica Sur Clinic & Foundation, Mexico City, Mexico; ^3^ Gastroenterology and Obesity Unit, Medica Sur Clinic & Foundation, Mexico City, Mexico

**Keywords:** liver steatosis, metabolic syndrome, endoscopy, surgery, bariatric

## Abstract

Nowadays, non-alcoholic fatty liver disease is one of the first causes of liver transplant worldwide; many efforts have been done to find the perfect drug for this multifactorial disease. Presently we just have a few drugs that could be used in specific and limited clinical scenarios. Current evidence suggests that bariatric endoscopic and surgical therapies could be strategies with optimal outcomes, with high impact in quality of life, decrease of cardiovascular risk, and improvement in metabolic profile, despite being considered expensive procedures. This review proposes to consider these therapies early together with liver fibrosis evaluation, with long term cost-effectiveness benefits in the absence of response to lifestyle modifications and pharmacological treatments.

## Introduction

As time passes by, our lifestyle changes and we put health aside. With the increased prevalence of obesity and weight-related metabolic comorbidities (MC), non-alcoholic fatty liver disease (NAFLD) has become one of the most common causes of chronic liver disease and the major cause of liver transplant. Worldwide prevalence is about 25%, with median age estimated in 50 years (1), with geographical and ethnic differences; most notably a protective effect on black ethnicities and conversely higher rates of non-alcoholic steatohepatitis (NASH) in Hispanic groups, perhaps partially secondary to higher frequency of genetic risk variants. The highest prevalence has been reported in South America and the Middle East (2–4).

NAFLD exhibits a spectrum of histologic features that includes steatosis, non-alcoholic steatohepatitis (NASH), fibrosis, cirrhosis, and hepatocellular carcinoma (HCC) (5). High-calorie diets, excess of refined carbohydrates, sugar-sweetened beverages, and high fructose intake have all been associated with weight gain, obesity, and NAFLD (6, 7). NAFLD is generally observed in overweight and obese patients with MC and it is considered the hepatic manifestation of metabolic syndrome; however, NAFLD with normal weight is defined as lean NAFLD. In these patients, the etiology of liver disease seems to be more related to genetic dysfunctions than to lifestyle (8). Even though NAFLD was considered a part of metabolic syndrome and obesity, the guidelines of the American Association for the Study of Liver Diseases recommend NAFLD screening in all patients with DM and those with at least two metabolic abnormalities; suspicion of NASH should be stronger in DM patients specifically (6).

NAFLD physiopathology is multifactorial, with impairments in multiple metabolic pathways; nonetheless, all of these lead to triglycerides accumulation in hepatocytes, triggering inflammation and activation of hepatic stellate cells. One third of patients with early-stage NASH will progress to fibrosis within 5 to 10 years after the clinical diagnosis is made (9).

Despite the pharmacological efforts to obtain an effective molecule for NAFLD treatment, lifestyle modifications focused on weight loss and sedentarism avoidance are the first-line and optimal treatment for these patients nowadays. Weight loss is the cornerstone for improvement and prevention of NAFLD progression; a 3-5% body weight loss achieves a significant reduction of steatosis and intrahepatic lipid content, whereas a 7-10% body weight loss could improve histological parameters such as steatosis, inflammation, and fibrosis; up to 10% of body weight loss is needed to improve necroinflammation (6, 10).

Richard L. Varco and Henry Buchwald published a book entitled Metabolic Surgery, in which they define this phase of surgical evolution as “the operative manipulation of a normal organ system to achieve a biological result for a potential health gain” (11). Since 1991, bariatric surgery (BS) is still one of the most effective and successful methods for producing weight loss in obese patients (12, 13). Over time, benefits of BS were observed in comorbidities associated to obesity, mainly improvement of hyperlipidemia (60-90%) and improvement or resolution of diabetes mellitus (DM) (74-93% and 47-98%, respectively) (14). Regarding liver disease, BS has demonstrated resolution of NASH and progressive reduction of fibrosis in long term follow-up (15); moreover, and compared with non-surgical treatments, BS has been associated with lower cardiovascular risk, which is the most common cause of death in NAFLD patients (6, 16). However, outcomes are metabolic in these studies, none of which contemplates NAFLD resolution as the main outcome.

The aim of this review is to present current evidence of endoscopic and surgical bariatric procedures in NAFLD patients and evaluate them as an additional early therapeutic option, together with lifestyle modifications and pharmacological treatments.

## Treatment options for NAFLD

### Lifestyle modifications

Lifestyle modifications are the first-line treatment option for NAFLD (17); goals of these modifications are focused on preventing the progression of liver disease to fibrosis or HCC and treating MC through weight loss with diet and exercise. Independently of the type of diet, a minimum of 3%-5% weight loss is necessary to improve steatosis; this reduction should be accomplished by a combination of hypocaloric diet with daily reduction of 500-1000 kcal and physical activity (6). It has been observed that 150 minutes of moderate-intensity aerobic exercise per week could decrease cardiovascular risk (18). Resistance exercise in combination with aerobic physical activity decreases the risk of sarcopenia (19).

A recent network meta-analysis concludes that current evidence shows considerable uncertainty about lifestyle interventions and modifications in NAFLD patients; clinical trials should align more closely to the standard clinical practices in order to evaluate direct and indirect effects of interventions; moreover, it is necessary to evaluate mortality, quality of life, cirrhosis decompensation, cost-effectiveness, and transplant as endpoints, with adequate and sufficient follow-up to establish significant clinical effects of interventions related to diets and physical activity (20).

### Pharmacological treatment

There are several pharmacological therapies proposed for NAFLD and NASH patients with different mechanisms of action. The optimal pharmacological therapy for NAFLD should reduce steatosis, inflammation, and fibrosis in order to prevent progression of liver disease to cirrhosis or HCC; additionally, improvement of metabolic background and prevention of DM and cardiovascular disease should be goals for new molecules, understanding the metabolic complexity of fatty liver disease (21). Different current treatment guidelines consider insulin sensitizers, glucagon-like peptide 1 analogues, and antioxidants as therapy adjuvants for NAFLD patients; however, once again, due to the complex physiopathology of NAFLD, there is not a single and ideal pharmacological therapy at the time.

Metformin is an insulin sensitizer with the ability to decrease gluconeogenesis in the liver and to increase fatty acid oxidation in adipose tissue. Metformin has been studied in non-diabetic NAFLD patients, showing improvement in liver biochemistry and HOMA-IR index, in combination with dietary treatment (21); however, metformin was not recommended for NAFLD or NASH treatment since there is no evidence of significant improvement of liver histology (6).

Pioglitazone is an oral hypoglycemic drug from the thiazolidinediones family, which are ligands for the nuclear transcription factor peroxisome proliferator-activated receptor gamma, with broad effects on glucose and lipid metabolism, as well as on vascular biology and inflammation. The mechanism of action is the increase of insulin sensitivity through the activation of the gamma isoform of peroxisome proliferator-activated receptor. This activation includes fatty acid transporter protein, lipoprotein lipase, glucokinase, and the GLUT4 glucose transporter, which helps to reduce IR in muscle, liver, an adipose tissue (22). On the other hand, vitamin E is an antioxidant that has proven to decrease aminotransferases in NAFLD patients and to improve steatosis, inflammation, and ballooning in non-diabetic NAFLD patients (23). Vitamin E has three main effects: 1) antioxidant by increasing enzymatic antioxidant activity and genetic modulation, and by decreasing apoptosis and fibrogenesis; 2) anti-apoptotic by decreasing pro-apoptotic enzymes (BAX and P53); and 3) anti-inflammatory by decreasing inflammatory cytokines, such as TNF-a and interleukins.

The effects of combining vitamin E and pioglitazone have been observed. Serum alanine and aspartate aminotransferase (AST, ALT) levels were reduced with vitamin E with pioglitazone, as compared to placebo (p<0.001 for both comparisons), and both agents were associated with reductions in hepatic steatosis (p= 0.005 for vitamin E and p<0.001 for pioglitazone) and lobular inflammation (p= 0.02 for vitamin E and p= 0.004 for pioglitazone), but without improvement in fibrosis scores (p= 0.24 for vitamin E or p= 0.12 for pioglitazone) (23). Another study observed that vitamin E decreases death and transplant risk (HR 0.30, CI95% 0.12 - 0.74, p<0.01) (24). Despite these results, pioglitazone was not recommended for NAFLD patients and vitamin E is only recommended for non-diabetic NASH patients; none of these agents should be used in patients without histological evidence of NASH or NAFLD (6). However, the safety of vitamin E has been analyzed, showing an increase in overall mortality (25), hemorrhagic cerebrovascular events incidence (26), and prostate cancer (27). Given that the treatment with vitamin E should be administrated by long periods of time, these findings need to be considered and doses should be adjusted for each patient.

GLP-1 agonists are incretin hormones derived from the gut. They are a relatively novel class of antidiabetic medications. Native GLP-1 lowers blood glucose by inducing insulin secretion and reducing glucagon secretion. Liraglutide is the most widely studied medication (17). A multicenter, double-blind, randomized, placebo-controlled phase 2 study demonstrated a statistically significant resolution of steatohepatitis without worsening fibrosis, which was the primary endpoint comparing liraglutide vs placebo. The use of liraglutide has shown hepatocyte ballooning score p=0.05, steatosis p=0.0009, lobular inflammation p=0.65, glucose p=0.005, insulin p=0.91, and homeostatic model of insulin resistance (HOMA-IR) p=0.23. However, there was no statistically significant change in lobular inflammation and NASH (28).

In a recent Phase II clinical trial, the effect of GLP1 receptor agonist semaglutide for NASH resolution was evaluated. Patients with a 0.4 mg dose of semaglutide showed 59% of NASH resolution with no worsening of fibrosis, with a mean 13% of weight loss compared to placebo. Improvement in the fibrosis stage has not been observed (29). Results of Phase III clinical trials that evaluate the effect of semaglutide and its adverse effects, such as nausea and constipation, are required in order to position this therapeutic option for NAFLD patients. At the time, evidence of GLP-1 antagonist is premature to consider it as a NAFLD or NASH treatment; in a recent meta-analysis, Dutta et al. concluded that the observed improvements in ALT and some imaging features with low doses of semaglutide are limited for recommendation due to the small number of patients who were evaluated (6, 30).

Recent evidence suggests that sodium-glucose cotransporter 2 in (SGLT2) inhibitors suppresses the development of NAFLD in humans. Yoshimasa et al. studied 63 NAFLD patients with type 2 DM, at least 20 years old, with a glycated hemoglobin level of 6.0%-12.0%. This study was performed according to a prospective, randomized, and open-label design. The study showed no significant decreases in the SDPP-4 serum level together with liver enzymes (AST, ALT, and gamma glutamil transaminase GGT) in this type of patients. Changes in clinical parameters were AST, p= 0.3353; ALT, p= 0.4493; GGT, p=0.4584; and NAFLD fibrosis score, p= 0.5225. However, the study has some limitations; for example, serum sDPP-4 was not well balanced between the groups at the baseline, since serum sDPP-4 was somewhat higher in the SGLT2 (dapagliflozin) group than in the control group (31).

Farsenoid X-activated receptor (FXR) is related to metabolic stress pathways, energy expenditure, and lipogenesis control; therefore, it has been proposed as a pharmacological option to improve insulin sensitivity in NAFLD and DM patients (32). Obeticholic acid, an agonist of FXR activity, has been studied for NAFLD treatment in FLINT (33) and REGENERATE (34) studies, showing an improvement of fibrosis with a 25 mg dose, compared to placebo in Phase III interim analysis; however, differences are not clinically significant enough; adverse events incidence needs to be taken into account, as well as Food and Drug Administration warnings related to other liver diseases (35).

Despite the existence of different pharmacological options, some of them have not shown to be superior to lifestyle modifications and some others have shown adverse effects or have not achieved the main aims; therefore, the best options today are pioglitazone and vitamin E, with limitations.

## Bariatric surgery in NAFLD

There is solid evidence about the efficacy of BS in patients with morbid obesity through different surgical techniques, such as classic bypass, adjusted gastric band (AGB), and gastric sleeve gastrectomy (SG); according to guidelines, patients eligible for BS are those with a body mass index (BMI) ≥40 kg/m2; patients with BMI ≥35 kg/m2 with one or more obesity-related complications, majorly metabolic, including evidence of NAFLD/NASH, and patients with BMI 30-34.9 kg/m2 and DM with inadequate glycemic control; however, B and C are recommended for patients with BMI 30 to 35 GRADE (36).

BS seems to be the most effective treatment for obesity with a profound effect in MC (37). Due to this, BS has been proposed as an indication focused on improving metabolic outcomes (metabolic surgery) for non-morbid obese patients. In three systematic reviews of 16 randomized clinical trials, a decrease in blood pressure in patients who underwent bariatric procedures (Roux-en-Y gastric bypass (RYGB), AGB, and SG) has been observed; nonetheless, and despite the results in favor of metabolic surgery, these studies have low methodological quality and the evidence has very low certainty, where only hypertension was evaluated as comorbidity (38). More studies are needed in order to evaluate bariatric procedures in non-morbid obese patients with metabolic outcomes as the major aim.

Documented by a routine liver biopsy of BS, it has been reported that NAFLD is present in >95% of patients who undergo bariatric procedures (9); due to this, the benefits of BS in liver outcomes, majorly steatosis and fibrosis, have been studied with different evaluation methods such as pre and post-surgery biopsies, non-invasive diagnostic methods, and serum markers.

Evidence about the benefits of BS in NAFLD is relatively lower than other obesity comorbidities such as DM, and once again, evaluations of liver outcomes are heterogeneous. An analysis in 2017 demonstrated an increase in life expectancy and quality-adjusted survival after surgery in patients with all classes of obesity (39), where BS showed a significant weight lost effect. It has the potential to stop the progression of NAFLD by decreasing liver inflammation and fibrosis. A prospective study by Vilar-Gómez et al. suggested that the amount of weight loss is correlated with the degree in histologic improvement of liver disease (10).

Regarding steatosis evaluation by liver biopsy, one of the most important studies was performed by Caiazzo et al, who evaluated 413 perioperative and five-year after BS liver biopsies, finding a significative improvement of NAFLD Activity Score (NAS) after AGB (1.7 ± 1.4 vs 1.0 ± 1.3, p <0.001) and RYGB (2.0 ± 1.5 vs. 0.7 ± 1.2, p <0.001) (40). In a more specific histology evaluation, Praveen et al. evaluated 30 paired biopsies in 7.1 months follow-up time, and they reported that 19 patients have steatosis resolution, 12 lobular inflammation resolution, and 1 patient presented fibrosis improvement (p<0.05 for all) (41).

NAFLD progression to fibrosis is the most important outcome in this liver disease; therefore, the evaluation of fibrosis resolution is highly relevant in BS. According to this, a prospective study demonstrated the changes compared to baseline three and six months after surgery, showing a regression of steatosis and fibrosis measured with OwLiver (p = 0.002), Fibrotest (p = 0.061), SteatoTest (p = 0.0001), NASHTest (p = 0.0002), and Fatty Liver in ultrasound (p = 0.008) (42). Nickel et al. evaluated liver fibrosis improvement in 100 patients scheduled for laparoscopic sleeve gastrectomy or RYGB, with transient hepatic elastography and BARD score (BMI, aspartate aminotransferase (AST), alanine aminotransferase (ALT), and DM); one year after BS, liver stiffness showed a significant improvement (12.9 ± 10.4 vs. 7.1 ± 3.7 kPa, p <0.001) as well as in BARD score (2.3 ± 1.2 vs. 2.8 ± 1.1, p = 0.008); however, a BARD score above 2 points represents a high risk for advanced fibrosis (43). Despite these favorable results, is important to consider that came from majorly of non-invasive biomarkers.

BS has demonstrated benefits in NASH and liver fibrosis related to weight loss and metabolic improvement; however, the exact mechanisms of regression for these entities have not been fully elucidated (44). Monitoring of NASH and fibrosis patients who underwent BS is necessary to adjust risk and long-term treatments; noninvasive methods have been proposed as useful follow-up tools (45). As for cost-effectiveness, BS was effective in NAFLD patients regardless of their fibrosis stage, with an increase in quality adjusted-life years (39). This benefit is due to a metabolic impairment control. A retrospective cohort study analysis evaluates the progression of NAFLD to cirrhosis. It includes 115,374 patients with NAFLD diagnosis from 2003 to 2015, 2942 of whom subsequently underwent BS and were compared versus the non-surgical disease-control cohort. The median follow-up time was 32.3 months for BS patients and 31.3 months in the nonsurgical population; at the time of their surgery, up to 80% of BS patients demonstrated histologic findings of NAFLD and 15-30% had evidence of NASH or fibrosis. The results of the analysis showed that BS was associated with a significant risk reduction of cirrhosis in NAFLD patients (HR 0.31, 95% Cl 0.19.0.52, p <0.001) compared to those without surgical treatment, with a median follow-up time of 31 months (46).

BS has been reported to lead to complete resolution of NAFLD, following the sustained weight loss induced in obese patients; evidence comes from studies with different outcomes such as resolution of obesity and improvement of metabolic diseases. On the other hand, for specific strong liver outcomes, Barreto de Brito e Silva et al. evaluated the impact of BS on NAFLD and NASH in 45 studies with biopsy assessment. They observed improvement in NASH and fibrosis in patients who underwent RYGB and SG. These beneficial results have also been observed in biochemical liver parameters (47). In a recent meta-analysis of 37 studies with histological evaluations, Zhou et al. observed a significant decrease in risk of steatosis, ballooning degeneration, inflammation, and fibrosis (48). Despite the heterogeneity of outcomes and measurements, different systematic reviews and meta-analyses have been performed in order to evaluate the effect of BS in NAFLD. Apparently, these therapeutic options are effective in improving histological (steatosis, steatohepatitis, and fibrosis) and biochemical liver parameters. (**Table 1**)

**Table 1:** Results of meta-analysis of bariatric procedures in non-alcoholic fatty liver disease.

Author	Year	Aim	Number of studies	Principal results
Bower (49)	2015	Quantify the effects of bariatric surgery on changes in NAFLD liver histology and biochemistry	29	**Histology** • Steatosis: WMDI 50.2% (95%CI 35.5–65.0) • Lobular Inflammation: WMDI 50.7% (95%CI 26.6–74.8) • Hepatocyte Ballooning: WMDI 67.7% (95%CI 56.9–78.5) • Fibrosis: WMDI 11.9% (95%CI 7.4–16.3) **Biochemistry** • ALT WMR 11.63 u/l (95%CI 8.34–14.39) • AST WMR 3.91 u/l (95%CI 2.23–5.59) • ALP WMR 10.55 u/l (95%CI 4.40–16.70) • GGT WMR 18.39 u/l (95%CI 12.6224.16)
Fakhry (9)	2019	Evaluate the impact of surgically induced weight loss on histologic and biochemical features of NAFLD.	21	**Histology** (pooled proportion of patients with improve) • Steatosis: 88% (95% CI: 0.80-0.94) • Steatohepatitis: 59% (95% CI: 0.38 -0.78) • Fibrosis: 30% (95% CI: 0.21 -0.41) **Biochemistry** (pooled proportion of patients with improve) • AST 32% (95% CI:0.22-0.42) • ALT 62% (95% CI: 0.42 - 0.82) • ALP 45% (95% CI: 0.19- 0.71)
Lee (50)	2019	Establish the harms and benefits of bariatric surgery on histologically confirmed resolution of NAFLD (steatosis, inflammation, ballooning degeneration, and fibrosis), NAS, and histologic worsening of NAFLD.	32	• Steatosis ES 0.66 (95% CI 0.56-0.75) • Inflammation ES 0.50 (95% CI 0.35-0.64) • Ballooning degeneration ES 0.76 (95% CI 0.64-0.86) • Fibrosis ES 0.40 (95% CI 0.29-0.51) • NAS MD 2.39 (95% CI 1.58-3.20) • Histologic worsening of NAFLD ES 0.12 (95% CI 0.05-0.20)
Barreto de Brito e Silva (47)	2021	Evaluate bariatric surgery’s impact on NAFLD and NASH using histologic and serum biochemical criteria and compares the difference between RYGB and SG on NAFLD/NASH postoperative improvement.	45	Histology • Steatohepatitis • RYGB RD 0.53 (95% CI 0.33-0.74) • SG RD 0.42 (95% CI 0.27 – 0.57) • Fibrosis • RYGB RD 0.26 (95% CI 0.14 – 0.37) • SG RD 0.20 (95% CI -0.00 – 0.39) • NAS • RYGB RD 2.52 (95% CI 1.50 – 3.55) • SG RD 2.25 (95% CI 1.96 – 2.54) • Grade Fibrosis • RYGB RD 0.77 (95% CI 0.49 – 1.05) • SG RD 0.76 (95% CI 0.59 – 0.93) • Biochemical • AST • RYGB MD 5.15 (95% CI 2.39 – 7.91) • SG MD 8.02 (95% CI 5.80 – 10.25) • ALT • RYGB MD 12.04 (95% CI 8.44 – 15.65) • SG MD 15.43 (95% CI 12.99 – 17.86) • ALP • RYGB MD 4.47 (95% CI -3.44 – 12.59) • SG MD 13.75 (95% CI 3.08 – 24.43) • GGT • RYGB MD 19.06 (95% CI 13.77 – 24.34) • SG MD 12.27 (95% CI 8.40 – 16.15) **Histological RYGB vs SG** • NAS MD -0.09 (95% CI -2.34 – 2.16) • Grade of fibrosis MD -0.53 (95% CI -1.78 – 0.72) **Biochemical RYGB vs SG** • AST MD 0.66 (95% CI -1.53 – 2.86) • ALT MD 5.54 (95% CI 2.85 – 8.23) • ALP MD18.38 (95% CI 8.52 – 28.23) • GGT MD -2.58 (95% CI -22.36 – 17.19)
Zhou (48)	2022	Evaluate the effect of bariatric surgery on histologically proven remission of NAFLD (steatosis, inflammation, ballooning degeneration, and fibrosis) and NAS	37	• Steatosis RD -0.56 (95% CI -0.69, -0.42) • Ballooning degeneration RD -0.49 (95% CI -0.61, 0.37) • Inflammation RD -0.44 (95% CI -0.58, -0.30) • Fibrosis RD -0.25 (-0.32, -0.18) • NAS MD 0.95 (95% CI 0.17 – 1.74)
Ren (51)	2022	Evaluate the effect of endoscopic bariatric and metabolic therapies on NAFLD patients with obesity	33	**Steatosis** • CAP by TE MD -53.76 (95%CI -73.04, -34.47) • HSI MD -5.25 (95%CI -8.39, -2.11) • NAS MD -3.00 (95%CI -3.27, -2.73) **Biochemical** • ALT MD -12.44 (95%CI -14.70, -10.19) • AST MD -7.88 (95%CI -11.11, -4.64) • GGT MD -12.07 (95%CI -15.79, -8.35) • TG MD -0.33 (95%CI -0.43, -0.22) • HOMA-IR MD -1.9 (95%CI -2.49, -1.30) **Fibrosis** • NAFLD Score MD -0.58 (95%CI -0.97, -0.20) • Liver stiffness by TE MD -6.39 (95%CI -13.73, 0.96) • Fib4 Index MD -0.28 (95%CI -0.63, 0.07)

NAFLD non-alcoholic fatty liver disease; NASH non-alcoholic steatohepatitis; WMDI weighted mean decrease in incidence; WMR weighted mean reduction; AST aspartate aminotransferase; ALT alanine aminotransferase; ALP alkaline phosphatase; NAS NAFLD activity score; ES effect size; MD mean difference; RD risk difference; RYGB Roux-en-Y gastric bypass; SG sleeve gastrectomy.

As for the surgical technique, a 5-year controlled longitudinal study compares the benefit of RYGB versus AGB on NAFLD; at baseline, NAFLD was present in 86% of the patients and was categorized as severe (NAS ≥3) in 22%. RYGB patients had a higher BMI (49.8 ± 8.2 vs 46.8 ± 6.5 kg/m2, p<0.001) and more severe NAFLD (NAS 2.0 ± 1.5 vs 1.7 ± 1.4, p = 0.004) than AGB patients. Weight loss after 5 years was 25.5% ± 11.8% after RYGB vs 21.4% ± 12.7% after AGB (p<0.001). When analyzed with a mixed model, all NAFLD parameters improved after surgery (p<0.001) and improved significantly more after RYGB than after AGB (% steatosis: 1 year, 7.9 ± 13.7 vs 17.9 ± 21.5, p<0.001; 5 years, 8.7 ± 7.1 vs 14.5 ± 20.8, p<0.05; NAS: 1 year, 0.7 ± 1.0 vs 1.1 ± 1.2, p<0.001; 5 years, 0.7 ± 1.2 vs 1.0 ± 1.3, p<0.05). In a multivariate analysis, the superiority of RYGB was primarily but not entirely explained by weight loss (40). Regarding the long term effectiveness of SG in a recent, but small cohort in Japan, Murakami et al. observed maintenance of body weight loss and decreased liver enzymes and steatosis in liver biopsies in patients with SG; however, an improvement in liver fibrosis has not been observed (52).

In a recent hierarchical network meta-analysis, Panunzi et al. showed in 48 studies, comparing different pharmacological options and BS, that pioglitazone and RYGB are the most effective treatments for reducing NAS (-1.50 (95% CrI -2.08, -1.00)) for pioglitazone and -1.00 (95% CrI -1.70, -0.32) for RYGB. Despite being quality evidence, it is necessary to consider that the analysis was focused on NASH and liver fibrosis only, the lack of randomized clinical trials for BS, and that the size of the effect could be affected by low power observed in some studies (20).

One anastomosis gastric bypass (OAGB) is a relatively new bariatric procedure that is a modification of biliopancreatic diversion with a duodenal switch that confers less malabsorptive components. This technique was approved by expert consensus in 2018 as a safety standard bariatric procedure (53). OAGB has been evaluated in NAFLD patients; one of the first studies is a case report by Motamedi et al. (54); they observed a progression of NAFLD, evaluated by liver biopsy, after a rapid weight loss by OAGB in a female patient. Later, Kermansaravi et al. evaluated weight loss and obesity-related comorbidities in 24 patients who underwent OAGB; 73% of those patients show a complete remission of NAFLD 12 months after surgery, in addition to remission of other metabolic outcomes such as DM, hypertension, and dyslipidemia (53). Salman et al. (55) evaluated the long-term effects of OAGB on biochemical, clinical, and histopathological liver outcomes in 67 morbid obese patients; after 15 months, patients show a significant decrease in liver function test, as well as in NAS Score components such as steatosis, ballooning, and lobular inflammation. OAGB could be a promising technique for NAFLD patients, although more evidence is necessary to evaluate safety, benefits, and complications in these patients.

## Bariatric endoscopic techniques in NAFLD

Endoscopic bariatric and metabolic therapies (EBMT) are an alternative option for patients; however, evidence about the effectiveness of these techniques is still insufficient, particularly for liver disease. EBMT are: intragastric balloon (IGB); endoscopic sleeve gastroplasty (ESG); aspiration device; transpyloric shuttle; Botox injection; duodenal jejunal bypass liner (DJBL); duodenal mucosa resurfacing (DMR); and incisionless partial jejunal diversion for primary obesity surgery endoluminal (POSE). These techniques are focused on weight loss, but metabolic effects such as improved glycemic control, lipid profile, and cardiovascular markers have also been observed in a mean of 12 months of use (56). Evidence of EBMT comes from observational studies, mostly with aims related to obesity and DM; the effects of these therapies on NAFLD parameters is an unclear and poorly studied area; transpyloric shuttle and Botox injection have not been evaluated for NAFLD or liver parameters.

The effect of the IGB on gastric emptying is one of the many processes by which hunger and satiation are modulated. The first mechanistic study demonstrated that the IGB produced delays in gastric emptying, compared to lifestyle interventions alone (57). The IGB has gained popularity during the last few years, but it is not considered an effective treatment in the long-term. Lee et al. have assessed changes in liver histology and showed a significant reduction of NAS and a decrease in BMI, AST, and ALT after 6 months. IGB has been proposed as a tolerable and potentially effective procedure as initial treatment for morbid obesity before a definitive surgical procedure, with a weight loss mean of 21.2 ± 14kg, majorly in patients with BMI>50 kg/m2. This preoperative procedure could decrease risks of BS in “super obese” patients (58); however, the preoperative IGB did not show effectiveness in postsurgical morbidity (59).

Another technique of this kind is the ESG, which consists in gastric volume reduction performed with an endoscopic suturing device (60). In a study of 91 patients, reduced levels of HbA1c, systolic blood pressure, triglycerides, and ALT 12 months after ESG were observed, suggesting improvements in metabolic dysfunction and liver steatosis, but liver outcomes have not been evaluated as a major aim (61).

DMR has been proposed as an endoscopic procedure to treat patients with DM and NAFLD (62). The procedure involves the circumferential hydrothermal ablation of the duodenal mucosa to allow for its regeneration. The procedure includes marking the location of the papilla of Vater and inserting a guidewire past the ligament of Treitz. The catheter is pushed over the guidewire to produce submucosal expansion in order to provide a protective layer of saline between the mucosa/submucosa and the proper duodenal muscle layer, as well as a stepwise circumferential hydrothermal ablation at 90°C (63).

A single-center study in Santiago, Chile, performed a 6-month follow-up of 85 patients with DM who received endoscopic DMR treatment. Safety was assessed in all patients. Efficacy was evaluated in patients who received at least 9 cm of duodenal ablation (n=67). Endpoints included HbA1c, fasting plasma glucose, weight, and AST. A metabolomic analysis was conducted in a subgroup (n = 14). HbA1c was lower 6 months after DMR than at baseline (7.9 ± 0.2% vs. 9.0 ± 0.2%, p<0.001). Fasting plasma glucose was also significantly lower 6 months after DMR compared to baseline (161 ± 7 mg/dl vs. 189 ± 6 mg/dl, p=0.005). Body weight decreased slightly. Six months after, ALT had decreased from 41 ± 3 IU/L to 29 ± 2 IU/L (p <0.001) and AST had decreased from 30 ± 2 IU/L to 23 ± 1 IU/L (p<0.001). The metabolomic analysis demonstrated that DMR had key lipid-lowering, insulin-sensitizing, and anti-inflammatory effects, as well as an increasing antioxidant capacity. Mean FIB-4 had also markedly decreased (64).

Recently, the REVITA-1 study results associated DMR with long term improvements in insulin sensitivity and other glycemic parameters, such as HbA1c, after 24 months in DM patients; although liver parameters are not a specific outcome, a decrease in ALT and triglycerides levels was observed (65); with these promising results in metabolic parameters, studies about the benefits of DMR on NAFLD are needed to recommend this endoscopic technique as a therapeutic option.

In the FIH study, hydrothermal ablation was successfully administered with no evidence of perforation, pancreatitis, or hemorrhage. Duodenal biopsy specimens obtained 3 months after the procedure demonstrated full mucosal regrowth. No inflammation was observed, and there was minimal-to-mild collagen banding deposition observed in a proportion of ablation site biopsy specimens, with no evidence of fibrotic scarring. Glycemic and hepatic measures improved over a 6-month follow-up (63).

As for endoscopic DJBL, a decrease in liver biochemistry (AST and GGT) has been observed in patients with DM and obesity after six months of having received the device; six months after the removal of the device, only ALT decrease is maintained. This endoscopic procedure has been evaluated for ten years in retrospective and prospective, but noncomparative, studies with a 12-month follow up, with a sample size of 16 to 61 patients, majorly obese and obese with DM. Their liver outcomes are biochemical parameters since there are no main aims; however, an improvement in ALT, AST, GGT, and triglycerides levels has been observed (51).

As mentioned before, the evidence about the effectiveness of EBMT in specific NAFLD outcomes is poor. A recent meta-analysis evaluated the effects of EBMT on obese NAFLD patients; the results showed that endoscopic procedures significantly improved liver steatosis and decreased biochemical levels; regarding fibrosis, the effect of EBMT was observed only when this pathology was evaluated by NAFLD-Score, but no significant differences were observed in other measurements such as elastography (**Table 1**); nonetheless, authors conclude that large scale, prospective, and long-term studies are needed to clarify the role and recommendation of endoscopic procedures in NAFLD patients (51).

Studies with an adequate sample size and aims focused on liver biomarkers are needed; currently only two studies of IGB evaluate liver histology as an outcome (66, 67); most studies only evaluate subrogate NAFLD parameters such as biochemistry or non-invasive methods and blood scores for liver fibrosis. However, beneficial effects on liver steatosis could be achieved since other metabolic parameters are improved, such as glycemic and lipid control.

## Limitations, complications, and mortality of BS

The perioperative mortality of BS is estimated in 0.08%. The postoperative complications are associated with obesity. In the immediate postoperative period, the most common complications are bleeding, infections, and thromboembolisms. The most common post-surgery complication is peritonitis due to the formation of an anastomotic fistula, with a 1-6% incidence after gastric bypass and 3-7% after SG. Other surgical complications include fistula, bleeding, herniation, gastric erosion, and small bowel obstructions. Iron deficiency (49-50%) (68) and protein malnutrition has been observed in patients after BS, being protein deficiency a potentially serious complication (69). Post-operative malnutrition is extremely rare and it is due to the restriction and change in absorption (70).

As for maintenance, it has been estimated that up to 30% of patients with unsuccessful bariatric procedures experienced insufficient weight loss (71) or weight gain up to 50%. Weight loss failure is defined as insufficient weight loss 18 months after surgery and progressive weight regain after successful weight loss (72). Weight regains are due to different causes, such as type of surgery, increased ghrelin levels, and inadequate follow-up support or maladaptive lifestyle behaviors. A systematic review of studies with patients who underwent SG showed regain rates from 5.7% at 2 years to 75% at 6 years (73). However, there are no adverse effects of BS in NAFLD reported at the moment.

Regarding adverse effects of EBMT, in a 21 studies meta-analysis, Ren et al. reported nausea, vomiting, and abdominal pain as common adverse events, with incidence of serious adverse events of 0% to 19%; however, reports of evaluated studies were inconsistent and fragmented (51).

## Future perspectives

Current evidence shows that NAFLD is the epidemic that we are and will be facing in the next decades. Lifestyle modifications are considered the cornerstone for NAFLD treatment and, unfortunately, we have failed in obtaining a specific treatment. Since NAFLD and obesity share many metabolic pathways, it is practically impossible to make a single molecule hit many targets. It has been estimated that the prevalence of liver cirrhosis secondary to NAFLD will have a considerable increase in the years to come.

There is a specific group of patients that do not meet the current recommendations for BS, but have MC refractory to conventional treatment or even liver fibrosis; in these patients, surgical indications could be modified to become more inclusive in order to avoid the development or aggravation of comorbidities in NAFLD patients, and morbid obesity; on the other hand, patients with BMI between 30 – 35 kg/m2 could be candidates for EBMT. This could be a bridging therapy; once weight loss or improvement of MC have been accomplished, lifestyle modifications could be the single therapy; however, more evidence is still needed in the NAFLD scenario.

Most studies consider MC and BMI for bariatric surgical or endoscopic procedures; however, few studies involve liver fibrosis evaluation. Liver fibrosis has a key role in NAFLD progression; consequently, we consider that including liver fibrosis evaluation in decision algorithms is essential in patients with MC.

**Figure 1:**
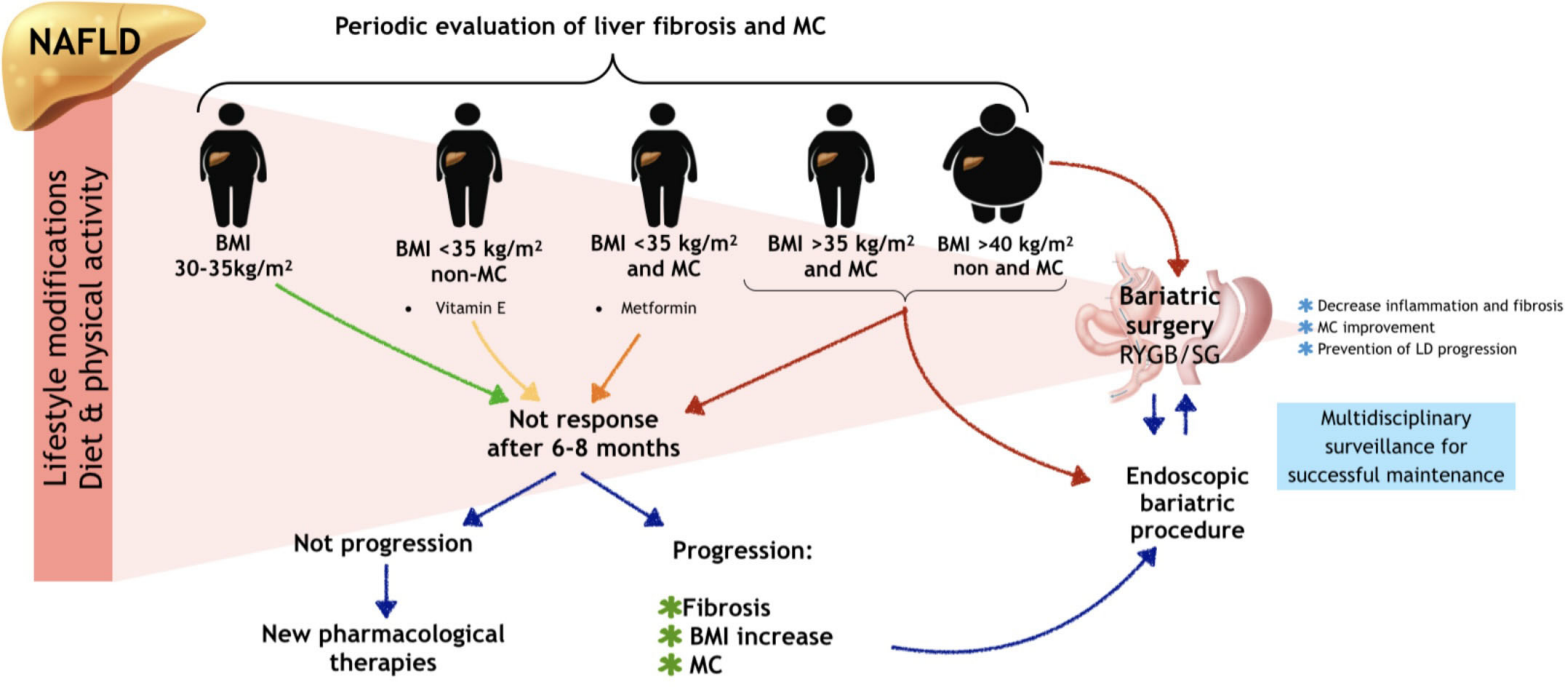
Fibrosis evaluation and NAFLD treatment in obese patients. NAFLD, non-alcoholic fatty liver disease; BMI, body mass index; MC, metabolic comorbidities; LD, liver disease. Patients who no response of first line of treatment (lifestyle modifications) after 6-8 months should be evaluated for progression of liver disease; in patients without progression new pharmacological therapies could be considered; in patients with liver disease progression endoscopic bariatric and metabolic therapies could be considered as an initial bariatric intervention, as well as in patients with BMI > 40 kg/m2. In all obese patients, liver fibrosis and metabolic comorbidities should be evaluated periodically. Lifestyle modifications should be at all time intervention in NAFLD patients.

In our proposal for NAFLD patients, the clinical approach must include a multidisciplinary team formed by an hepatologist, a gastroenterologist, a nutritionist, psychological support, an endoscopist, and a surgeon. At the beginning, all NAFLD diagnosed patients should be guided through the best evidenced first-line treatment (lifestyle modifications), which must be maintained constantly. Since MC and DM are strongly associated with worsening and progression of liver disease, patients should be evaluated for liver steatosis and fibrosis, ideally by non-invasive methods (elastography by magnetic resonance, transient elastography with control attenuated parameter). The fibrosis evaluation results would allow for an accurate identification of those patients with higher risk of liver disease progression. (**Figure 1**).

**Figure 2:**
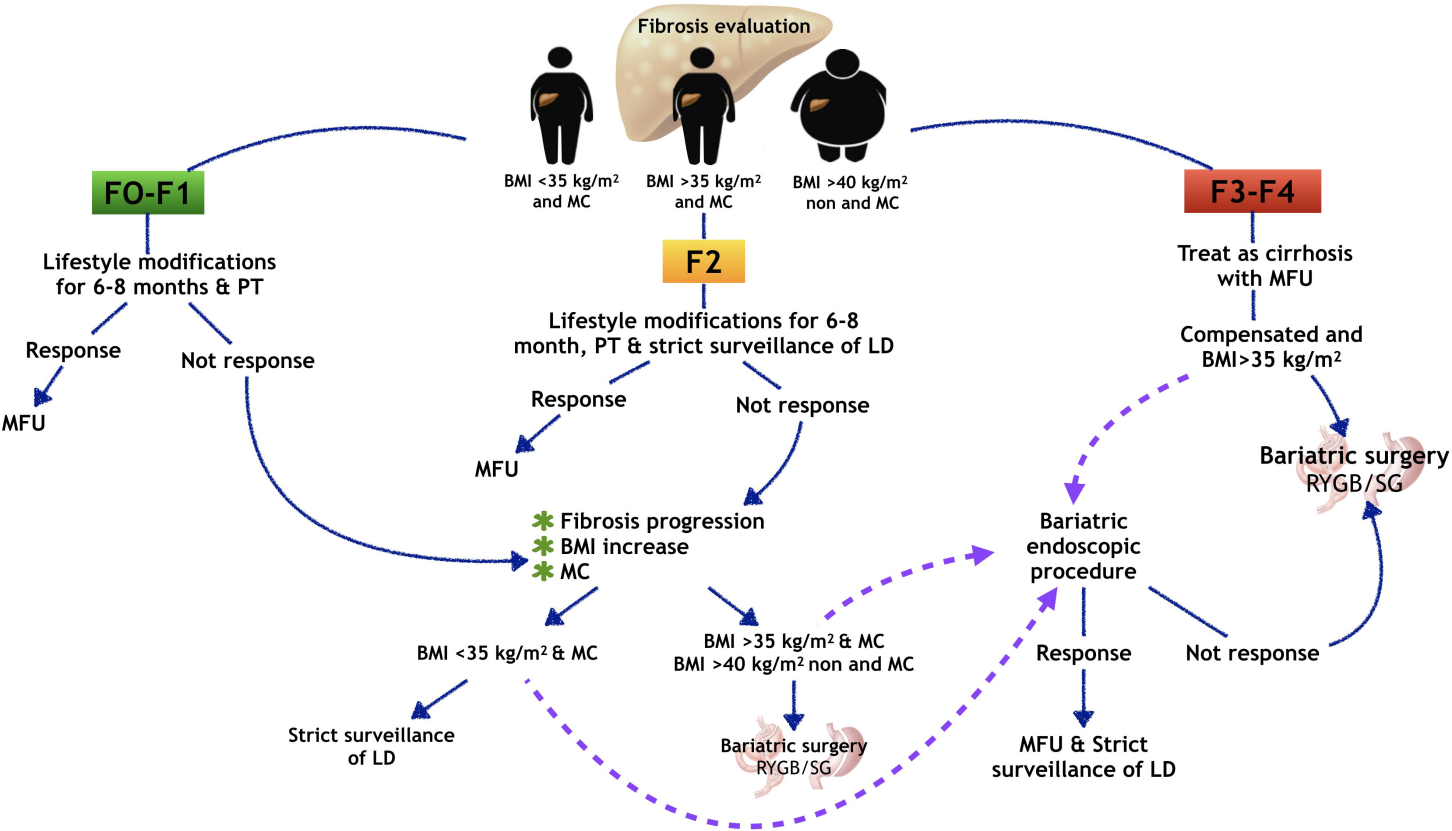
Liver fibrosis evaluation and bariatric procedures in patients with NAFLD. MFU, multidisciplinary follow-up; LD, liver disease; BMI, body mass index; MC, metabolic comorbidities; PT, pharmacological therapy; RYGB, Rux-en-Y gastric bypass; SG, sleeve gastrectomy. According to liver fibrosis evaluation in NAFLD patients, in those with F1-F2 lifestyle modifications with optional additional pharmacological therapy are recommended for 6-8 months. Patients with positive response should be continued with medical follow-up. Those patients with non-response and presenting liver fibrosis progression and worsening of BMI and MC could be scheduled to BS. Patients with advanced fibrosis should be treated as cirrhotic; in compensated patients with BMI>35 kg/m2, bariatric surgery could be recommended. The purple dotted lines representing our proposal of evaluating an endoscopic bariatric procedure as bridging therapy before BS, taking to account that more and better evidence are necessary to recommend endoscopic procedures in these patients.

**Figure 3:**
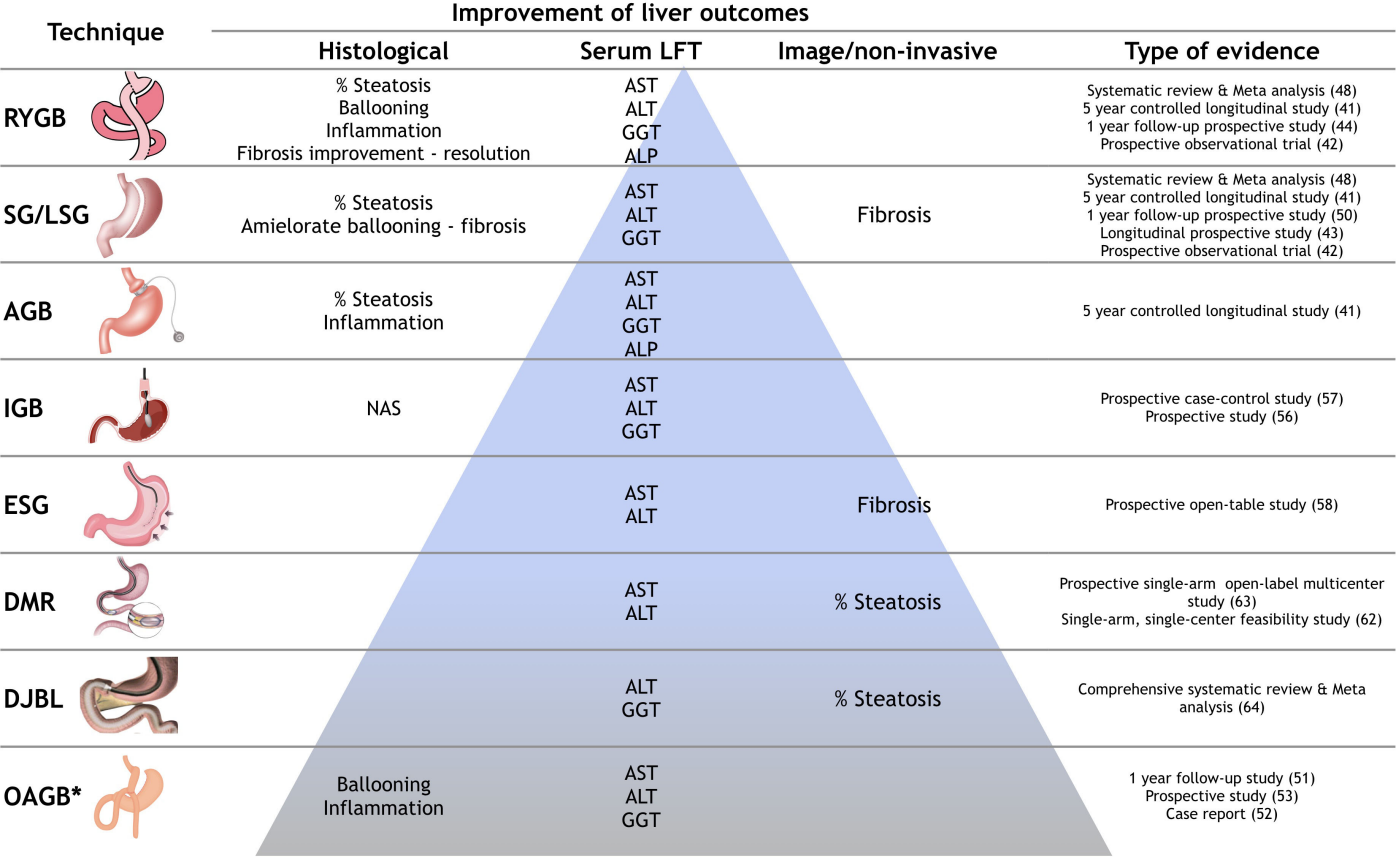
Evidence of effect of bariatric endoscopic and surgical procedures in NAFLD outcomes. Beneficial effects on liver outcomes of surgical and endoscopic procedures, according to level evidence (pyramid). LFT, liver function test; RYGB, Roux-en-Y gastric bypass; SG, sleeve gastrectomy; LSG, laparoscopic sleeve gastrectomy; AGB, adjusted gastric band; IGB, intragastric ballon; ESG, endoscopic sleeve gastroplasty; DMR, duodenal mucosa resurfacing; DJBL, duodenal jejunal bypass liner; OAGB, one anastomosis gastric bypass; AST, aspartate aminotransferase; ALT, alanine aminotransferase; GGT, gamma-glutamyl transpeptidase; ALP, alkaline phosphatase. *Results of 3 studies.

NAFLD patient assessment must always include monitoring of regression or progression of BMI, MC and, as mentioned before, liver fibrosis; hence we propose an algorithm for NAFLD patients that includes liver fibrosis evaluation. In those patients with F1, follow-up could be laxer than in patients with significant fibrosis (F2 to F4). There are no determining recommendations about follow-up timing in these patients currently, so we suggest that patients with weight loss, MC improvement, and no worsening of liver fibrosis could be followed-up periodically; those patients without progression but without improvement in lifestyle could be candidates for new pharmacological therapies. Metabolic endoscopic-surgical therapies could benefit higher-risk patients requiring close surveillance. Despite the first line interventions, weight and metabolic improvements are not achieved and fibrosis shows progression in these patients. (**Figure 2**) According to evidence, although EMBT seem to be attractive therapies due to their less invasive nature, surgical interventions such as gastric bypass have shown better outcomes; however, more evidence is necessary to support the EMBT efficacy. (**Figure 3**)

In patients with cirrhosis and obesity or MC, there are specific guidelines that determine the best time to offer and weigh the benefits of bariatric treatment. BS could be an option in order to prevent the progression of liver disease or complications, since patients with cirrhosis and excess of visceral fat have an increased risk of mortality, bacterial infections, sepsis-related death, and poor survival after liver transplant; therefore, weight loss, focused in sustained loss of excess body fat, is an important goal for these patients; however, the benefit of BS in cirrhotic patients has only been observed and recommended in patients who cannot achieve weight loss with lifestyle interventions and patients in compensated stages of cirrhosis in which evaluation of clinically significant portal hypertension is mandatory to schedule any elective surgical procedure (74). There is a lack of evidence regarding EBMT in these patients.

There are several gaps to recommend bariatric endoscopic/surgical procedures in NAFLD patients, such as adequate selection of characteristics of the ideal patient in terms of BMI, liver disease status and MC, metabolic criteria that could not fit the current BS recommendations in general population, and the fact that bariatric procedures are invasive and expensive treatments. In a not so far future, BS could be considered as an alternative therapeutic option in NAFLD patients, with different criteria to recommendation regarding liver fibrosis and risk of progression; on the other hand, taking into account the accelerated prevalence increase in liver diseases associated to metabolic dysfunctions, cost-benefit and cost-effectiveness are more points to be considered when including bariatric procedures in NAFLD treatment algorithms; however, we must always keep in mind that treatment success depends on maintaining the lifestyle modifications. The weakest part of the treatment in these patients is the high percentage of weight gain and the failure in lifestyle modifications; hence, close multidisciplinary surveillance plays a key role in NAFLD patient care.

There is no specific recommendation regarding NAFLD patient follow-up at the time, mainly due to the lack of evidence from real-life cohort studies. The TARGET-NASH study (75) is the first 5-year longitudinal prospective study that evaluates the effectiveness of treatment and follow-up in NAFLD patients, but the results have not been published yet. However, results of different observational studies have shown that lifestyle modifications should be maintained at least for 6 months in order to achieve changes in behavioral patterns (76). Treatment response in NAFLD patients is heterogeneous and not all patients are ready for a total change of habits (77); therefore, therapeutic options and strategies for improving clinical outcomes should be individualized and adapted to the clinical and metabolic characteristics of each patient.

Robust evidence have shown that BS in NAFLD seems to be a promising option that could be included in treatment algorithms; nevertheless, recently, Pais et al. (78) performed one of the most important studies that evaluate regression of NASH and fibrosis in 196 NAFLD patients who undergoing to BS with meticulous histological assessment and a 6 years median follow-up; they observed that despite of attractive results or BS on MC (weight loss, hypertension, diabetes, dyslipidemia and obstructive sleep apnea) resolution, results are not similar in NASH and fibrosis regression.

Even though the histological response (inflammation/fibrosis), seems to be that there a group of patients (47%) without worsening of advanced fibrosis but neither regression or resolution. Liver fibrosis is a fundamental outcome in NAFLD treatment targets, therefore results of Pais et al. study leads to new questions about fibrosis resolution by BS, concluding that there are patients that despite the bariatric procedure they will not have a positive response on liver advanced fibrosis or maybe require a more extensive follow-up to evaluate the regression or resolution of fibrosis, specially patients who underwent to bariatric procedure different to gastric bypass or elder patients. These non-responder patients could be candidates to new pharmacological therapies aimed to fibrosis regression.

Nowadays, bariatric endoscopic and surgical techniques are not considered solid recommendations in NAFLD treatment; however, evidence of these options in NAFLD patients seems to be solid, mainly in gastric bypass (**Figure 3**) but despite the positive results, patients that present bariatric treatment failure must be remembered. Possibly with future new evidence, BS could be an additional treatment option for NAFLD patients; likewise, EBMTs have shown efficacy but evidence is limited, however being less invasive procedures it could be considered an option as well. More and better designed studies, with specific liver outcomes, are needed to support the inclusion of BS and EBMT in NAFLD treatment algorithms. In any case, early interventions are key in these patients, and attending physicians should not wait for the progression to morbid obesity or MC to offer bariatric procedures.

## Author contributions

Conceptualization: IL-M, EJ-H; GC-N; Methodology: IL-M, EJ-H, GC-N. Literature analysis: EJ-H, AA; Writing - original draft preparation: EJ-H, AA; Writing - review and editing: IL-M, EJ-H, GC-N, MU; Supervision: MU. All authors contributed to the article and approved the submitted version.

## Conflict of interest

The authors declare that the research was conducted in the absence of any commercial or financial relationships that could be construed as a potential conflict of interest.

## Publisher’s note

All claims expressed in this article are solely those of the authors and do not necessarily represent those of their affiliated organizations, or those of the publisher, the editors and the reviewers. Any product that may be evaluated in this article, or claim that may be made by its manufacturer, is not guaranteed or endorsed by the publisher.
